# Functional validation of *ZbFAD2* and *ZbFAD3* in the alkylamide biosynthesis pathway from *Zanthoxylum bungeanum* Maxim

**DOI:** 10.3389/fpls.2022.991882

**Published:** 2022-09-23

**Authors:** Jie Zhang, Zhaochen Wu, Nuan Han, Dongmei Wang

**Affiliations:** Shaanxi Key Laboratory of Economic Plant Resources Development and Utilization, College of Forestry, Northwest A&F University, Yangling, China

**Keywords:** zanthoxylum bungeanum maxim, alkylamides, biosynthesis, *ZbFAD2* and *ZbFAD3*, functional validation, fatty acid dehydrogenase

## Abstract

The spicy taste and medicinal properties of *Zanthoxylum bungeanum* are imparted by several alkylamides. Although most studies have focused on their isolation and identification, few have reported their biosynthesis pathways. Among the differentially expressed genes (DEGs) reported in the numerous varieties of *Z. bungeanum*, some might contribute to alkylamide biosynthesis. However, they are not yet functionally validated. The present study explored the function of two genes, *ZbFAD2* and *ZbFAD3*, in the alkylamide biosynthesis pathway, and their stable and transient expression in *Arabidopsis thaliana* and *Nicotiana benthamiana* were also analyzed. As compared with the wild-type (WT), the fatty acid content analysis indicated that *ZbFAD2*-*A. thaliana* transgenic seeds had lower oleic acid and higher linoleic acid contents, while the *ZbFAD3*-*A. thaliana* transgenic seeds showed lower linoleic acid and higher α-linolenic acid levels. Moreover, hydroxy-α-sanshool, a major alkylamide, was considerably higher in the *ZbFAD2*-*N. benthamiana* transgenic plants (0.2167 ± 0.0026 mg/g) than in the WT (0.0875 ± 0.0049 mg/g), while it was lower in the *ZbFAD3*-*N. benthamiana* transgenic plants (0.0535 ± 0.0037 mg/g). These results suggest that both *ZbFAD2* and *ZbFAD3* are vital alkylamide biosynthesis enzymes in *Z. bungeanum*. Our study not only helps to scale up the alkylamide production, but also establishes the role of the uncharacterized genes.

## Introduction

*Zanthoxylum bungeanum Maxim*. (genus: *Zanthoxylum*; family Rutaceae) has recently gained significant attention from researchers because of its applications in the pharmaceutical, food, and cosmetic industries (Sun et al., 2019). They are widely distributed in the tropical and subtropical regions, including China, Japan, Korea, India, etc (Sun et al., 2019). To date, over 250 species have been identified globally in this genus, with China harboring at least 45 species (Sun et al., 2019). It contains a variety of health-beneficial bioactive compounds. Numerous studies have focused on the chemical structure and bioactivity of different compounds, including flavonoids, alkaloids, amides, lignans, and coumarins. Among these, alkylamides are known for the pungent flavor and pharmaceutical properties of *Z. bungeanum*. Twenty-seven alkylamides have been identified and characterized as unsaturated fatty acid amides (Zhao et al., 2013). Alkylamides are formed through conjunction with phenylalanine or valine along with unsaturated fatty acids. These compounds have diverse properties such as antiviral, antifungal, antibacterial, antidiabetic, anti-inflammatory, analgesic, antinociceptive, antioxidant, antithrombotic, antimutagenic, anticancer, antimalarial, antitrypanosomal, psychopharmacological, androgenic, spermatogenic, and insecticidal. They also have valuable immunomodulatory and ethnomedicinal uses and are effective in the treatment of anesthesia, neurodegeneration, and skin disorders (Greger, 1984). Therefore, it is imperative to study the natural origin and production of alkylamides due to their numerous benefits in medicine, food, and cosmetics. To date, most studies have focused on the identification and isolation of these compounds, while very few studies have studied the molecular biology behind their biosynthesis. Deciphering the biosynthesis pathways will not only enhance the understanding of the mechanism of action, but also help to augment their production through metabolic engineering. To achieve this, it is necessary to elucidate the numerous biosynthesis pathways, primary precursors, and genes involved in alkylamide regulation and biosynthesis. Previously, a transcriptome analysis of three varieties of *Z. bungeanum* was carried out (Wu et al., 2020), and 49 genes were expressed differentially. These genes are speculated to have roles in the alkylamide biosynthesis pathways. Therefore, the present study aims to determine the role of the *ZbFAD2* and *ZbFAD3* genes in the alkylamide biosynthesis pathway.

*Z. bungeanum* seeds contain over 90% unsaturated fatty acids (Sun et al., 2016). These are essential fatty acids as they cannot be synthesized by the human body (Stoutjesdijk et al., 2000). Their α- linolenic acid and linoleic acid contents are as high as ~70%. Fatty acid desaturases are extremely important in alkylamide biosynthesis. They desaturate most glycerolipids in plants, either membrane-bound or soluble and are present in the endoplasmic reticulum (ER) and chloroplasts (Bryant and Mezine, 1999). *FAD2* and *FAD3* mostly desaturate extra-chloroplastic lipids that are found as integral ER membrane-bound proteins (Los and Murata, 1998). In the ER, *FAD2* destaurates oleic acid (18:1) into linoleic acid (18:2), while *FAD3* destaurates linoleic acid into γ-linolenic acid (C18:3, n6) (Bhunia et al., 2016). During fatty acid desaturation, stearoyl-ACP desaturase first converts stearic acid into oleic acid in advanced plants. Then, the fatty acid dehydrogenase converts the unsaturated bonds into an acyl chain at particular positions (Los and Murata, 1998; Bhunia et al., 2016). The sensation of pungency related to Z. bungeanum is conferred by aliphatic alkylamides with unsaturated fatty acid chains and N-terminal isobutyl structures. Numerous studies on alkylamides have demonstrated the taste and bioactivities of Z. bungeanum (Caterina et al., 1997; Story et al., 2003; Bhunia et al., 2016; Zhang et al., 2019). Hydroxy-ϵ-sanshool, hydroxy-α-sanshool, hydroxy-β-sanshool, hydroxy-γ-sanshool, bungeanool, and isobungeanool are the main sources of alkylamides in *Z. bungeanum*. However, hydroxy-sanshool has been reported to affect the intensity of pungency in *Z. bungeanum* (Jordt et al., 2004). The molecular mechanisms of some alkylamide bioactivities have been deciphered, e.g., they display anticancer properties by disrupting the mitochondrion-dependent apoptotic pathway, thereby inhibiting cell survival (Jordt et al., 2004; Sugai et al., 2005). These compounds also improve glucose and lipid metabolism by modulating the A δ mechanosensory nociceptor activity and the pathway of the adenosine monophosphate-activated protein kinase, thereby affecting diabetes (Tsunozaki et al., 2013). Hydroxy-sanshool has been shown to decrease the occurrence of skin wrinkles (Kawai et al., 2013). It can also improve memory and learning by modulating the brain’s neuronal terminals (Sugai et al., 2005; Riera et al., 2009).

Alkylamides are considered a combination of unsaturated fatty acids, valine, or phenylalanine. However, the alkylamide biosynthesis pathway, critical precursors, and their associated genes in *Z. bungeanum* are yet to be identified. Although alkylamide compounds have been proven to be the key components of *Z. bungeanum*, their biosynthesis pathways remain largely unknown. Hydroxyl-α-sanshool is considered an important alkylamide variant. Upon examining the transcriptomes of the three *Z. bungeanum* varieties with significantly different hydroxyl-α-sanshool levels, it was found that several DEGs were associated with the unsaturated fatty acid, valine, and leucine biosynthesis tended to affect alkylamide synthesis (Rong et al., 2016). Plants are the only organisms capable of *de novo* fatty acid synthesis in the plastids, which include acetyl-CoA, palmitic acid (16:0), stearic acid (18:0), and oleic acid (18:1△^9^). These are then further desaturated to form linoleic acid (18:2△^9,12^) and linolenic acid (18:3△^9,12,15^) *via* the prokaryotic pathway. However, the eukaryotic pathway occurs *via* the ER (Koo et al., 2007). In the ER, *FAD2* catalyzes the first step of the polyunsaturated fatty acid biosynthesis, converting oleic acid to linoleic acid and linolenic acid by incorporating a double bond between carbon 12 and 13 at the sn-1 and sn-2 sites in phosphatidylcholine. Additionally, omega-6 *FAD2* and omega-3 *FAD* (*FAD3*) convert oleic acid (C18:1Δ9) to linoleic acid, linoleic acid, and α-linolenic acid (C18:3Δ^9,12,15^). *FAD3*, a linoleate desaturase, regulates the amount of C18:3 in the seed oil by converting the phosphatidylcholine-bound linoleate to linoleate in the ER. However, there are limited studies on the relationship of biosynthetic pathways between polyunsaturated *FADs* and alkylamides in *Z. bungeanum*. Furthermore, the whole genome study of *Z. bungeanum* has shown that *ZbFAD2* and *ZbFAD3* are crucial genes in sanshool biosynthesis (You et al., 2015).

The present study aimed to functionally validate the *ZbFAD2* and *ZbFAD3* genes, as well as evaluate the correlation between unsaturated fatty acids and alkylamide compounds. The findings will provide a theoretical basis for the research on the cannabinoids biosynthesis pathway in *Z. bungeanum*. Furthermore, the molecular research on this pathway in *Z. bungeanum* provides technical support for the accumulation and directional regulation of the compounds in *Z. bungeanum*, which has both theoretical significance and economic value.

## Materials and methods

### Profiling of alkylamide compounds in different varieties of *Zanthoxylum bungeanum*

Three *Z. bungeanum* varieties with considerable differences in their alkylamide content, including Hancheng stingless, Fengxian Dahongpao, and Fugu Huajiao (Wu et al., 2020), were used for alkylamide profiling (**Figure S1**). In a previous study, transcriptome sequencing of all three varieties was conducted to study their gene expression profiles, and then correlated with the biosynthesis of alkylamide compounds (Wu et al., 2020). RT-PCR was used to confirm the expression of both *FAD3* (*c102796 graph_c0*) and *FAD2* (*c99494 graph_c0*) genes. The enzyme classes encoded by the *ZbFAD2* and *ZbFAD3* genes were identified using homologous sequence comparison (Wu et al., 2020).

### Bioinformatics analysis of *ZbFAD2* and *ZbFAD3*

The lengths of the open reading frames (ORF Finder program: https://www.ncbi.nlm.nih.gov/orffinder/) of both FAD genes were determined. *ZbFAD2* and *ZbFAD3* sequences were subjected to BLAST search using the Uniprot database. Based on the FAD protein sequences from 10 different plants, the phylogenetic tree of the target genes (*ZbFAD2* and *ZbFAD3*) and their homologous sequences were constructed using the MEGA software (Tamura et al., 2011). Phylogenetic trees were constructed from the *ZbFAD2* and *ZbFAD3* protein sequences. Online analysis tools like PROSITE (http://us.expasy.org/prosite/) and InterPro method (http://www.ebi.ac.uk/interpro/) were used to analyze the domains of the protein sequence of the gene sequence of interest.

### Analysis of the functions of *ZbFAD2* and *ZbFAD3* in *Arabidopsis thaliana*

The ORF coding regions of *ZbFAD3* and *ZbFAD2* were amplified from *Z. bungeanum* by PCR with the following M13-specific primers: forward: 5’-CCCAGTCACGACGTTGTAAAACG-3’; reverse: 5’-CAGGAAACAGCTATGAC-3’. The PCR products were digested with KpnI and BamHI, and then inserted into the 35S-pCAMBIA2300 vector. This construct was transformed into the *Agrobacterium tumefaciens* GV3101 strain containing the pSoup helper plasmid. This was then transformed into Arabidopsis ecotype Col-0 *via* Agrobacterium-mediated transformation (Clough and Bent, 1998). T1 seeds were plated on 1/2 Murashige and Skoog (MS) medium containing 1% sucrose and 50 mg/mL kanamycin, for the selection of transformants. Homozygous T3 seeds were sown on 1/2 MS medium and stratified at 4°C for 3 d. T3 seeds were germinated in a growth chamber under a 16-h light/8-h dark cycle at 22°C for an additional 7 d. Seven-day-old plants germinated on 1/2 MS medium were transferred into pots containing a soil mixture (vermiculite: nutrient soil = 4:1). After being grown under favorable moisture conditions for 14 d, the number of surviving plants was recorded. There were three replicated assays. After maturation, the seeds were collected, and the differences in fatty acid composition between WT and transgenic *A. thaliana* seeds were quantified using gas chromatography (GC). With glyceryl triheptadecanoate (C17:0) as an internal standard (Fan et al., 2019), the proportion of the corresponding fatty acids in the seed was determined based on the peak time for each fatty acid methyl ester in the GC. The significant differences between groups were compared using the *t*-test (*p < 0.05 and **p < 0.01).

### Analysis of the function of *ZbFAD2* and *ZbFAD3* in *Nicotiana benthamiana*

The ORFs of *ZbFAD3* and *ZbFAD2* were amplified from *Z. bungeanum* by PCR with the M13 specific following primers: forward: 5’-CCCAGTCACGACGTTGTAAAACG-3’; reverse: 5’-CAGGAAACAGCTATGAC-3’. The KpnI and BamHI digested PCR products were inserted into the 35S-pCAMBIA2300 vector. This construct was transformed into the Agrobacterium tumefaciens GV3101 strain containing the pSoup helper plasmid. To further verify the functions of *ZbFAD2* and *ZbFAD3* in the biosynthesis of alkylamide compounds, 35S-pCAMBIA2300-*ZbFAD2* and 35S-pCAMBIA2300-*ZbFAD3* were transformed into *N. benthamiana via* the injection method (Luo et al., 2022). Moreover, high-performance liquid chromatography (HPLC) was conducted to detect the differences in alkylamide levels between the transgenic and WT plants.

### RNA extraction and quantitative real-time PCR

Total RNA was isolated using the TransZol UP reagent (Transgen Biotech Co., Ltd., Beijing, China) according to the manufacturer’s instructions. First-strand cDNA was synthesized by reverse transcription of purified total RNA using FastQuant RT Kit (Tiangen, Beijing, China). The CFX96 Touch TM (BioRaD, USA) quantitative PCR instrument was used to carry out the quantitative real-time polymerase chain reaction assay. Reactions comprised a 20-μL volume containing 2 μL diluted cDNA, 1 uL forward primer, 1 uL revise primer, and 25 uL 5x UltraSYBR Mixture. Thermal cycler conditions were: 95°C for 10 min, followed by 40 cycles of 95°C for 10 s, 56°C for 30 s and 72°C for 32 s, and 72°C for 2 min. The specificity of each primer pair was verified by melting curve analysis. The *N. benthamiana* β-actin gene was used as an internal control. The 2−ΔΔCT method (Livak and Schmittgen, 2001) was used for quantification, and the variation in expression was estimated from three biological replicates. The primer pairs used for qRT-PCR analysis are listed in **Supplemental Table 2**.

### Fatty acid analysis of Arabidopsis seeds

Ten milligrams of transgenic seeds were put into 2 mL of 5% H_2_SO_4_ (v/v) in methanol for methylesterification, and added to each sample 0.25 mL of n-hexane and 0.25 mg of internal standard triglyceride heptadecacarbonate (C17:0). The methylesterification reaction was carried out at 80°C for 2 h. After cooling to room temperature, 1 mL of n-hexane and 2 mL of 0.9% NaCl solution were added. The mixture was then centrifuged at 5000 rpm for 10 min and the supernatant was aspirated for loading. In a gas chromatograph, the fatty acid methylation products were determined using a flame ionization detector (FID) equipped with an AT-FFAP column (0.25 mm × 30 m, 0.25 µm). The GC conditions of the evaporation chamber and FID were set to 260°C and 280°C, respectively. The temperature of the column oven was 160°C/min, then heated to 240°C at 4°C/min, and held at this temperature for 9 min. Using the high-purity helium as the carrier gas, the gas flow in the column was 1.46 mL/min and the split ratio was 20:1 (Fan et al., 2019). Three biological replicates were performed for each sample.

### High-performance liquid chromatography assays

Tobacco leaves were ground to powder, 2 mL of chromatographic methanol was added, and ultrasonic extraction was performed at 60°C for 1 h. After passing through a 0.22 um filter head, it was refrigerated at 4°C for use. Three parallel experiments were performed for each sample.

The mobile phase is comprised of water (A) and acetonitrile (B). Hydroxy-α-Sanshool Elution Program 0 - 20 min, B: 25% - 55%; 20 - 40 min, B: 55% - 90%; 40 - 45 min, 90% B kept constant, Hydroxy-α- Sanshool. The retention time of hydroxy-α-sanshool was 20.554 min. The injection volume was 20 μL, the mobile phase flow rate was 0.8 mL/min, and the detection wavelength was 254 nm (Chen et al., 2019).

## Results

### Profiling of alkylamide compounds in different varieties of *Zanthoxylum bungeanum*

**Figure 1:**
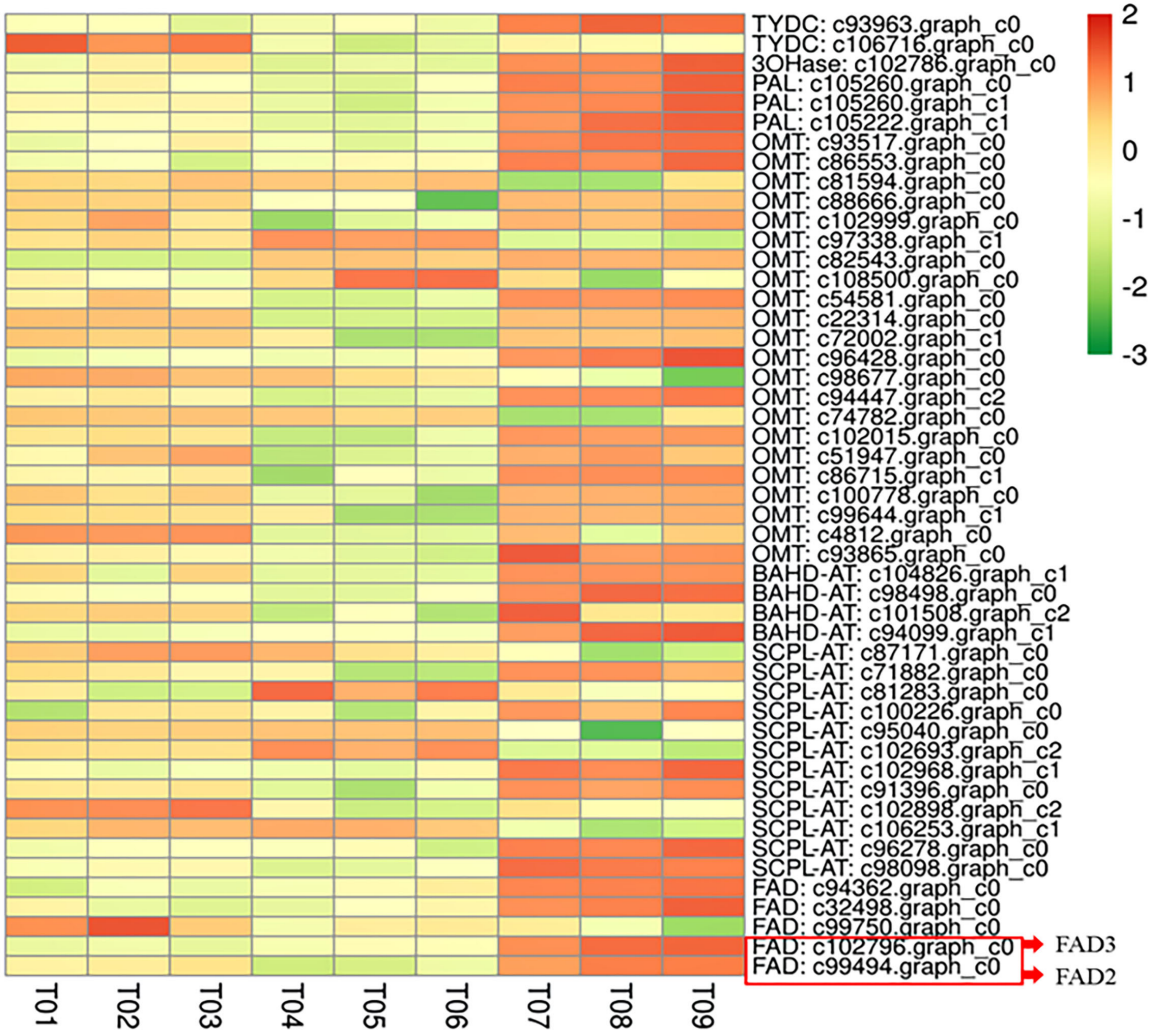
The expression levels of the DEGs related to the alkylamide biosynthesis pathway in *Z. bungeanum*. The highest expression of these genes was seen in Fugu (T07-T09), followed by Hancheng variety (T01-T03), with the lowest being in Fengxian (T04-T06).

No significant differences in the several morphological traits were found among the three varieties (**Figure S2**). All the candidate alkylamide biosynthesis genes were expressed in the three varieties, as shown in **Figure 1**. RT-PCR expression results revealed similar patterns in the DEG analysis. Among the candidate genes, several *FAD* genes showed obvious differences at the transcript levels, particularly the *c99494 graph_c0* and *c102796 graph_c0* sequences (**Table S1**). Our results are consistent with a previous study (Feng et al., 2021). Through homologous sequence comparison, we analyzed two full-length cDNA sequences in candidate *FAD* genes and identified them as unsaturated fatty acid dehydrogenases (now designated as *ZbFAD2* and *ZbFAD3*).

### Analysis of *ZbFAD2* and *ZbFAD3*

The ORFs of *ZbFAD2* and *ZbFAD3* were determined to be 1,167 bp and 1,152 bp long, respectively. The results showed that *Z. bungeanum* and *Citrus* were grouped together in the first place, however, farther than that between *Quercus* and *Jute*. An amino acid sequence analysis showed the following properties of *ZbFAD2* protein: (1) 383 amino acids long, (2) 44.16 kDa molecular weight, (3) isoelectric point of 8.04, (4) grand average of hydropathicity index (GRAVY) of −0.013, and (5) instability index of 37.48. Meanwhile, the properties of *ZbFAD3* protein were as follows: (1) 388 amino acids, (2) 44.79 kDa molecular weight, (3) isoelectric point of 8.76, (4) GRAVY of −0.116, and (5) instability index of 33.76 (**Figure S3**).

### Analysis of the functions of *ZbFAD2* and *ZbFAD3* in *Arabidopsis thaliana*

**Figure 2:**
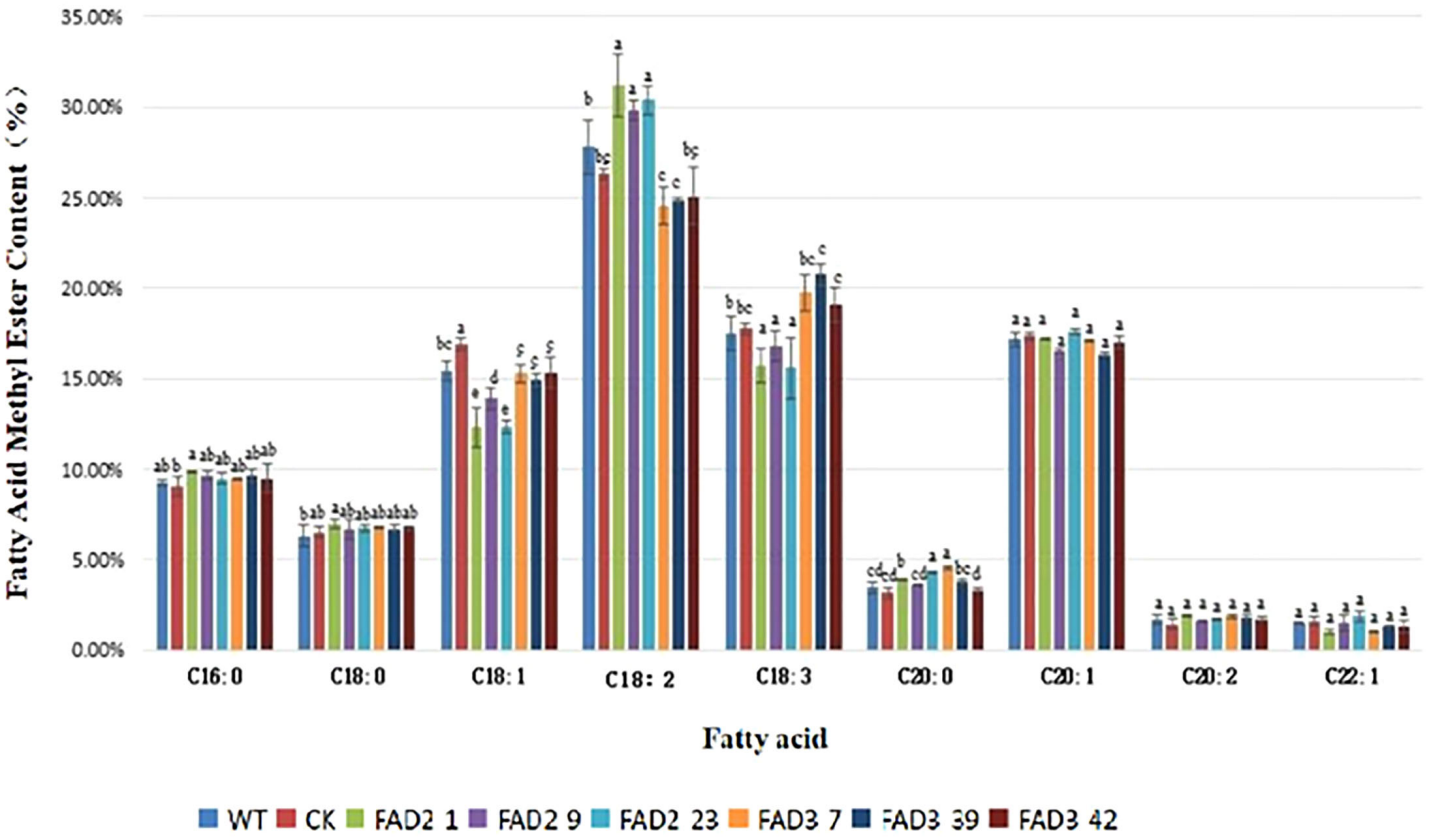
Fatty acid profiles and fatty acid methyl ester levels in the transgenic and WT *A. thaliana* seeds.

No apparent morphological difference was observed between the WT and transgenic *A. thaliana* plants grown up to 150 days. As shown in **Figure 2**, the level of linoleic acid was higher in the *ZbFAD2*-*A. thaliana* transgenic seeds (30.47%) than the WT (27.79%), while that of oleic acid was lower in the transgenic seeds (12.83%) than the WT (15.4%). However, the changes in the levels of other fatty acids were not significant. On the contrary, the level of α-linolenic acid was higher in the *ZbFAD3*-*A. thaliana* transgenic seeds (19.86%) than in the WT (17.47%), while that of linoleic acid was lower (24.84%) in the transgenic seeds than in the WT (27.79%). However, the changes in the levels of other fatty acids were also not significant in the *ZbFAD3*-*A. thaliana* seeds. Therefore, we demonstrated that the recombinant *ZbFAD2* and *ZbFAD3* proteins had desaturase activity and could catalyze the unsaturated fatty acid dehydrogenation. Thus, both recombinant *ZbFAD2* and *ZbFAD3* proteins could convert oleic acid into linoleic acid, and linoleic acid into α-linolenic acid, respectively, thereby playing an important role in the unsaturated fatty acid biosynthesis pathway.

### Analysis of the function of *ZbFAD2* and *ZbFAD3* in *Nicotiana benthamiana*

**Figure 3:**
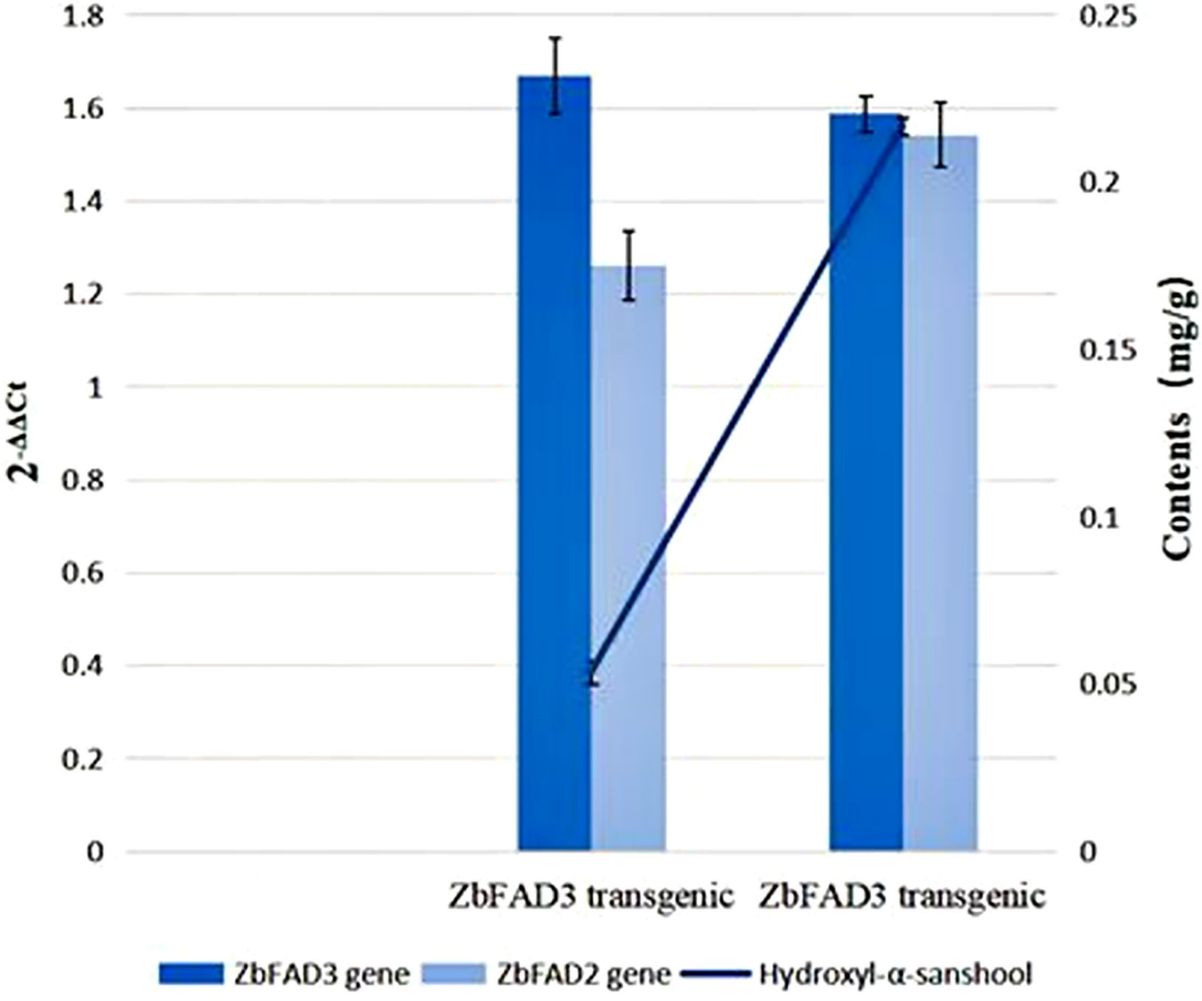
Expression analysis of the *ZbFAD*3 and *ZbFAD*2 genes using quantitative real-time PCR, of in the WT and transgenic *N benthamiana* plants.

We detected an increased expression for both *ZbFAD2* and *ZbFAD3* (**Figure 3**) *via* qRT-PCR assays on *ZbFAD3* and *ZbFAD2* injection method transgenic tobacco. Using the *N benthamiana ZbFAD3* and *ZbFAD2* gene as the internal reference gene, the relative expression levels of *ZbFAD3* and *ZbFAD2* genes in WT and two transgenic plants are shown in Fig 3, the results showed that the relative expression levels of these two genes were 10.69% and 17.45% higher in transgenic plants than WT, respectively. Simultaneously, the *ZbFAD3* transgenic plants showed that *ZbFAD3* gene expression amplified the expression of *ZbFAD2* gene. In contrast, an opposite trend was observed in the *ZbFAD2* transgenic plants, where the *ZbFAD2* gene expression reduced the expression level of *ZbFAD3* gene. Therefore, it shows that these two genes may affect cannabinoid synthesis by affecting the expression of other functional genes in the synthesis pathway.

**Figure 4:**
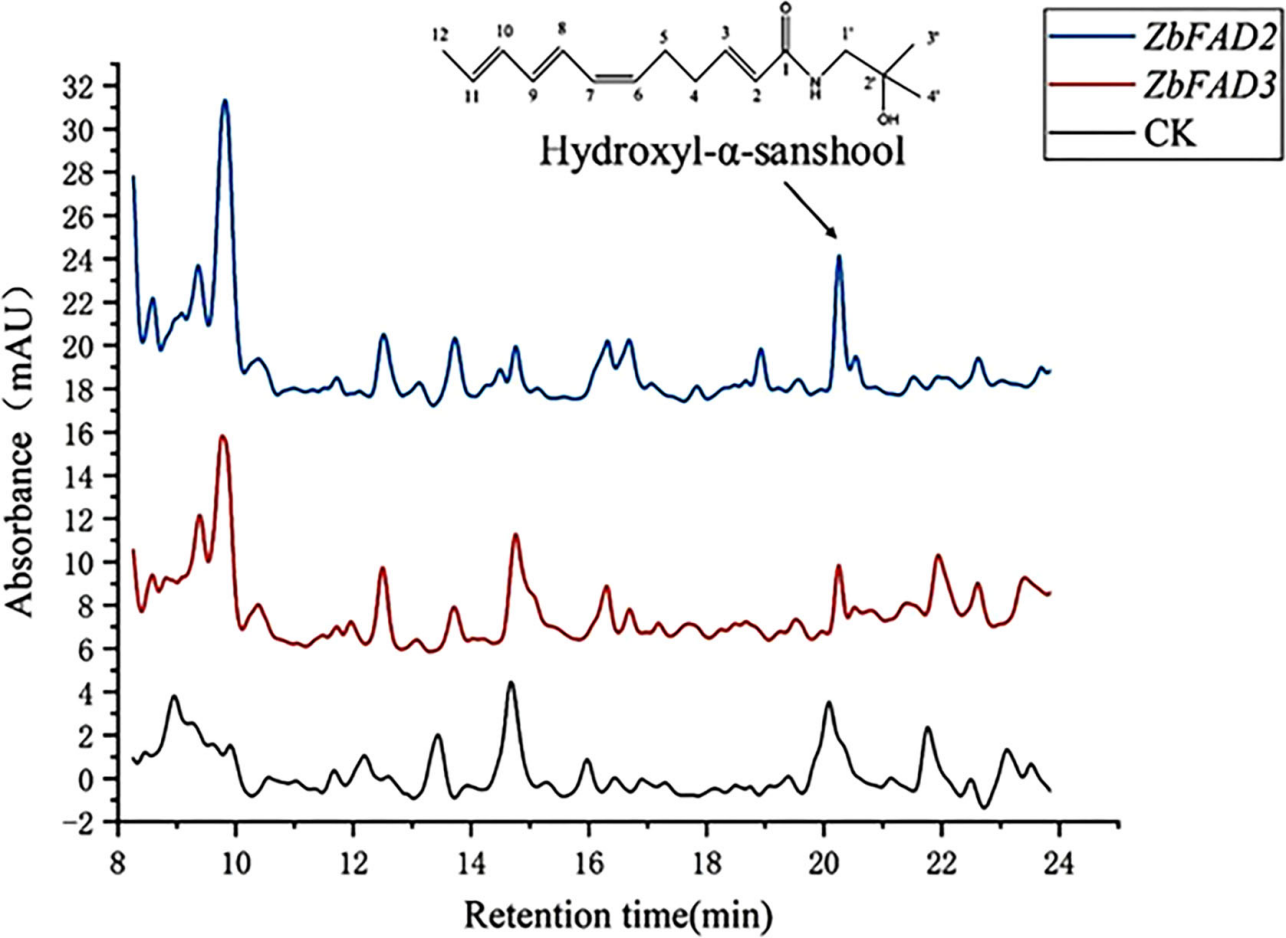
HPLC results of hydroxyl-α-sanshool in the *ZbFAD3-* and *ZbFAD2*-transgenic *N. benthamiana*.

The HPLC results indicated significantly higher hydroxy-α-sanshool levels in the *ZbFAD2*-transgenic *N. benthamiana* (0.2167 ± 0.0026 mg/g) plants as compared with the WT (0.0875 ± 0.0049 mg/g). Conversely, the level of hydroxy-α-sanshool was lower in the *ZbFAD3*-transgenic *N. benthamiana* (0.0535 ± 0.0037 mg/g) plants than in the WT (**Figure 4**). This is consistent with the previous findings that the expression of *ZbFAD3* was down-regulated and *ZbFAD2* was up-regulated in the transcriptome sequencing (Wu et al., 2020). Therefore, it can be inferred that these two candidate genes and unsaturated fatty acid biosynthesis are linked to alkylamide biosynthesis.

## Discussion

In recent years, *Z. bungeanum* has increasingly been proven to be a valuable source of bioactive compounds, which endowed it with several human health beneficial properties. Along with the pharmaceutical industry, both the cosmetics and food industries have also benefited. In this regard, numerous classes of compounds, including alkylamides, from *Z. bungeanum* have been explored and characterized. Alkylamides not only have an extensive range of bioactivities and applications, but also impart a distinct tingling sensation onto mucosal surfaces post application (Tsunozaki et al., 2013). Sanshools are valuable natural alkylamides that cause tingling and paresthesia when applied to the skin or tongue. These belong to a family of polyunsaturated fatty acid amides and constitute an essential flavor and medicinal ingredient in *Z. bungeanum*, which can be used as metabolic markers to evaluate *Z. bungeanum* quality. It is important to analyze different plant varieties, their gene expression, and the genetic differences in the *Z. bungeanum* biosynthetic pathways (Bhatt et al., 2017) for screening and improving its germplasm. Most studies have focused on their biological activities, extraction, and separation, but fewer have studied their secondary metabolism pathway and the synthetic regulation of sanshools. Therefore, the sanshool biosynthesis pathways and the associated active players involved in it still remain obscure (Cortez-Espinosa et al., 2011; Ren et al., 2017; Wu et al., 2020). The transcriptome sequencing of *Z. bungeanum* helped identify 19 DEGs related to unsaturated fatty acid, isoleucine, leucine, and valine biosynthesis. Keeping this in mind, our study results confirmed the importance of the *ZbFAD3* and *ZbFAD2* proteins in the alkylamide biosynthesis pathway.

This is the first time that *Arabidopsis thaliana* has been used to express the *ZbFAD2* and *ZbFAD3* proteins. Although *FAD2* and *FAD3*, which were previously thought to transform oleic acid into linoleic acid, had been studied in different plants, no reports were found on the cloning and subsequent characterization of these desaturases from *Z. bungeanum*. The current work is the first report on the isolation of *FAD2* and *FAD3* from this plant. We performed standard bioinformatics analyses to determine their role in the alkylamide biosynthesis pathways. Through phylogenetic analysis of both desaturases, and the results obtained from gene function verification, we found that these genes were closely associated with *PtFAD2* and *PeFAD2*. This will be helpful in managing the demand pressure of alkylamides in the medicine, cosmetics, and food industry (Xue et al., 2017; Gazave et al., 2020; Feng et al., 2021).

Both desaturases (*FAD2* and *FAD3*) are part of a larger family (FAD) of the membrane FADS-like superfamily. Five transmembrane helices, composed of a random coil, beta-turn, and alpha helix, were found in the secondary structures of *FAD2* and *FAD3*. According to the transcriptomic study of *Z. bungeanum*, *FAD5*, *FAD6*, *FAD7*, and *FAD8* were also involved in the biosynthesis of fatty acids. *FAD2* and *FAD6* converted oleic acid into linoleic acid *via* desaturation, whereas *FAD3*, *FAD7*, and *FAD8* converted linoleic acid into α-linolenic acid through desaturation. This highlights the potential of using *Z. bungeanum* for synthesizing linoleic acid (Chen et al., 2014; Busch et al., 2020). Increasing linoleic acid content in plants is mainly achieved through their trait-directed breeding. The expression of *FAD2* in Brassica napus and Brassica juncea increased their oleic acid content to 89% and 73%, respectively (Horiguchi et al., 2000). *FAD3* gene has been successfully cloned in Arabidopsis (Arondel et al., 1992), tobacco (Shimada et al., 2000; Orlova et al., 2003), wheat (Horiguchi et al., 2000), flax (Vrinten et al., 2005), rapeseed and other plants. It has been found that inhibiting the expression of *FAD3* in Arabidopsis thaliana or other plants, that is, inactivating and mutating it, will cause the content of 18:3 in the transgenic lines to decrease and the content of 18:2 to increase (Rahman et al., 1996). On the contrary, overexpression of *FAD3* gene will increase the 18:3 content in transgenic lines (Shimada et al., 2000; Orlova et al., 2003; Shah and Xin, 1997).

Ectopic expression of *FAD2* and *FAD3* genes in the *Arachis hypogaea*, *Sesamum indicum*, *Helianthusannuus*, etc different plants have shown that these genes could convert oleic acid into linoleic acid and generate hexadecadienoic acid (16:2 Δ^9,12^), using palmitoleic acid (16:1Δ^9^) as a catalyzed substrate. This may be attributed to some specific substrate preference or varied transmembrane topologies found in the *FAD2*s and *FAD3*s (Huang et al., 2010; Fan et al., 2019).

Three *Z. bungeanum* varieties were subjected to transcriptomic analysis in a previous study (Wu et al., 2020). Based on the obtained data, we found that all alkylamide biosynthesis-related genes were expressed in the three varieties, with varying degrees of expression. However, some *FAD* genes exhibited differences in transcript levels, which were consistent with previous findings. Upon performing a homologous sequence comparison based on the cDNA sequences of these *FAD* genes, we identified them as belonging to the unsaturated fatty acid dehydrogenase family and subsequently designated them as *ZbFAD2* and *ZbFAD3*. The lengths of the ORFs of these enzymes were determined to be 1,167 bp and 1,152 bp, respectively. By comparing the expression pattern and linoleic content, we found that the expression levels of *ZbFAD2* and *ZbFAD3* were positively correlated with the linoleic acid concentration of *Z. bungeanum*. The findings demonstrate that *ZbFAD2* and *ZbFAD3* significantly affect the accumulation and regulation of polyunsaturated fatty acids (PUFAs), by converting oleic acid to linoleic acid at different growth stages of *Z. bungeanum*.

Based on the results of bioinformatics analysis and prokaryotic expression, we found that *ZbFAD2* and *ZbFAD3* encoded ER-located-like *FAD2* and *FAD3* enzymes. The functional characterization of desaturases in *A. thaliana* tested the ability of *ZbFAD2* and *ZbFAD3* proteins in catalyzing linoleic acid synthesis. Our study provides proteomic data for the molecular regulation and coordination in *Z. bungeanum* with high linoleic acid content. Further characterization of *ZbFAD2* and *ZbFAD3* and their functional relationship may provide more information on the mechanisms controlling the fatty acid composition of *Z. bungeanum*, which may help in trait improvement *via* both genetic engineering and breeding.

The molecular weight of both purified proteins was very close to the bioinformatically-predicted value. Our findings showed that *ZbFAD2* reacted with oleic acid (C18:1△^9^) to produce linoleic acid (C18:2△^9,12^), while *ZbFAD3* potentially reacted with linoleic acid to produce α-linolenic acid (C18:3△^9,12,15^) (**Figure 4**). The homologous sequence analysis revealed that *ZbFAD2* and *ZbFAD3* could catalyze the conversion from oleic acid to linoleic acid, and the conversion from linoleic acid to α-linolenic acid, respectively. Therefore, both unsaturated fatty acid dehydrogenases function by introducing a second and third double bond to the fatty acid chain.

It was observed that the linoleic acid and oleic acid contents in the *ZbFAD2*-*A. thaliana* transgenic seeds were higher and lower than in the WT, respectively. However, the levels of α-linolenic acid and linoleic acid in the *ZbFAD3*-*A. thaliana* transgenic seeds were higher and lower than in the WT, respectively. Our results indicated that the recombinant *ZbFAD2* and *ZbFAD3* proteins functioned as desaturase enzymes and could catalyze unsaturated fatty acid dehydrogenation; which agreed with the prokaryotic expression analysis results, thereby confirming their roles in the unsaturated fatty acid biosynthesis pathway.

At present, both *FAD2* and *FAD3* genes have been successfully cloned in perilla, tobacco, Arabidopsis, wheat, flax, etc. The content of 18:3 in rice bran oil and roots increased significantly after the soybean *FAD2* and *FAD3* genes were transformed into rice, with the α-linolenic acid content in rice bran oil increasing significantly. These results show that overexpression of *FAD2* and *FAD3* genes can increase the α-linolenic acid content in plants. Therefore, we speculate that both *ZbFAD2* and *ZbFAD3* genes have an important role in the alkylamide biosynthesis pathway.

## Conclusion

To sum up, the prokaryotic and eukaryotic expression vectors of *ZbFAD2* and *ZbFAD3* genes were constructed, and their functions were evaluated *via* substrate reactions. Furthermore, expressing them in *A. thaliana* and *N. benthamiana* could identify their functional roles in the alkylamide biosynthesis pathway. The difference in the levels of unsaturated fatty acids and hydroxy-α-sanshool indicated that the two target genes were involved in the alkylamide biosynthesis pathway, and fatty acid synthesis was correlated with alkylamide synthesis. Our transient assays on *N. benthamiana* leaves and stable transformation on *A. thaliana* demonstrated that the expression of *ZbFAD2* and *ZbFAD3* genes induced the expression of some key genes for the biosynthesis of hydroxy-α-sanshool. It was observed that *Z. bungeanum* possessed an updated pathway for hydroxyl-α-sanshool biosynthesis (**Figure S4**). Therefore, *ZbFAD2* and *ZbFAD3* are the crucial enzymes for regulating the alkylamide biosynthesis pathway in *Z. bungeanum*. These would lay the groundwork for further research of the sanshools in the plants. Nevertheless, future studies should investigate the relationship between the biosynthesis of fatty acids and alkylamide compounds in *Z. bungeanum* to gain further insights into the alkylamide biosynthesis pathway.

## Data availability statement

The original contributions presented in the study are included in the article/**Supplementary Materials**. Further inquiries can be directed to the corresponding author.

## Author contributions

JZ performed the statistical analysis and drafted the manuscript. ZW and NH participated in the statistical analysis and the writing of the manuscript. DW conceived the research, participated in the research design and coordination, and provided suggestions on the writing of the manuscript. All authors contributed to the article and approved the submitted version.

## Funding

This work was supported by the National Natural Science Foundation of China (31872706), and the National Key Research and Development Program of China (2019YFD1000603).

## Conflict of interest

The authors declare that the research was conducted in the absence of any commercial or financial relationships that could be construed as a potential conflict of interest.

## Publisher’s note

All claims expressed in this article are solely those of the authors and do not necessarily represent those of their affiliated organizations, or those of the publisher, the editors and the reviewers. Any product that may be evaluated in this article, or claim that may be made by its manufacturer, is not guaranteed or endorsed by the publisher.
