# Supplementary material for: Functional validation of ZbFAD2 and ZbFAD3 in the alkylamide biosynthesis pathway from Zanthoxylum bungeanum Maxim
Source: Front Plant Sci. 2022 Sep 23;13:991882. doi: 10.3389/fpls.2022.991882 (PMC9563095; doi:10.3389/fpls.2022.991882)
Supplement: Supplementary file 1 [file Table_1.docx]

**Supplementary material**


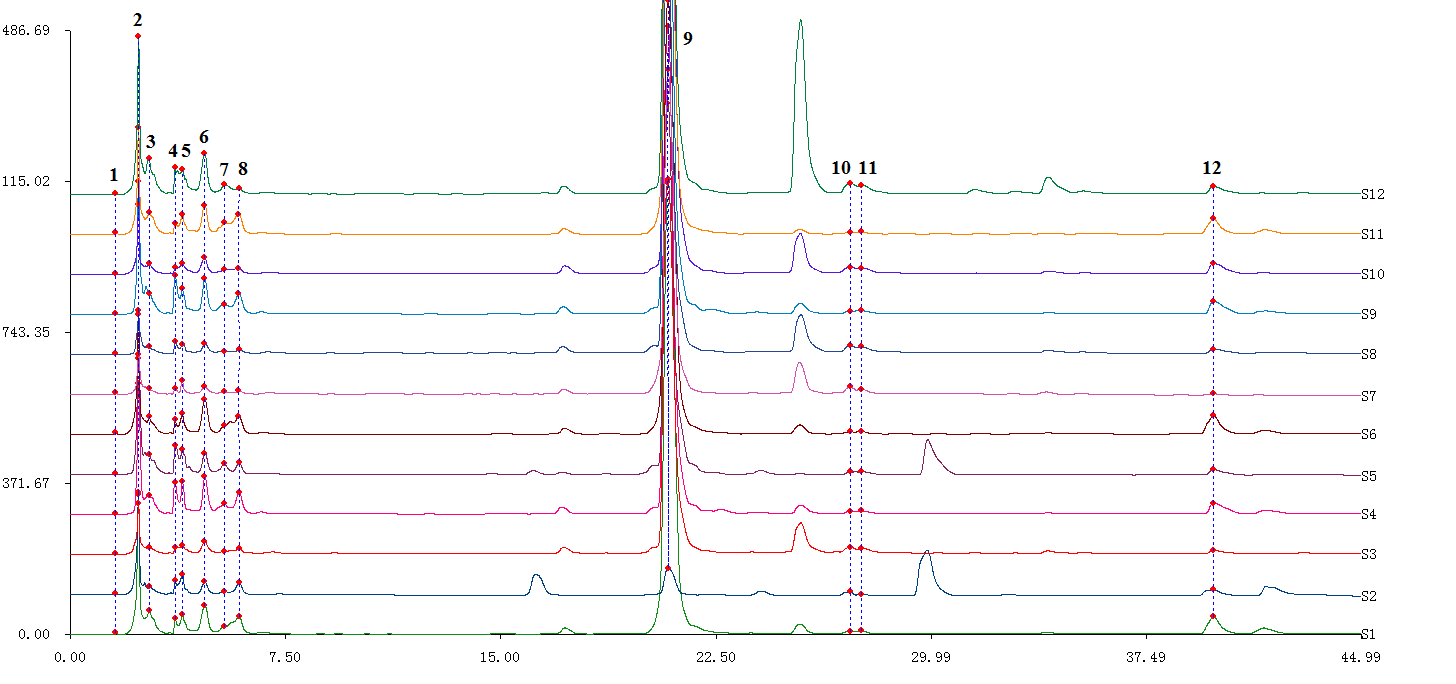


**Figure S1.** Comparison of the levels of the major alkylamide compounds in *Z. bungeanum*.


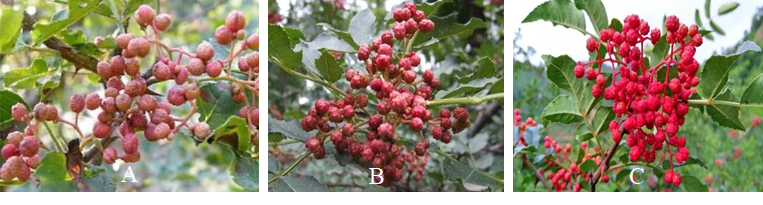


**Figure S2.** Characterization of the two genotypes of *Z. bungeanum* seeds. The seeds were randomly selected from Fugu (A), Hancheng (B), and Fengxian (C) *Z. bungeanum* plants.


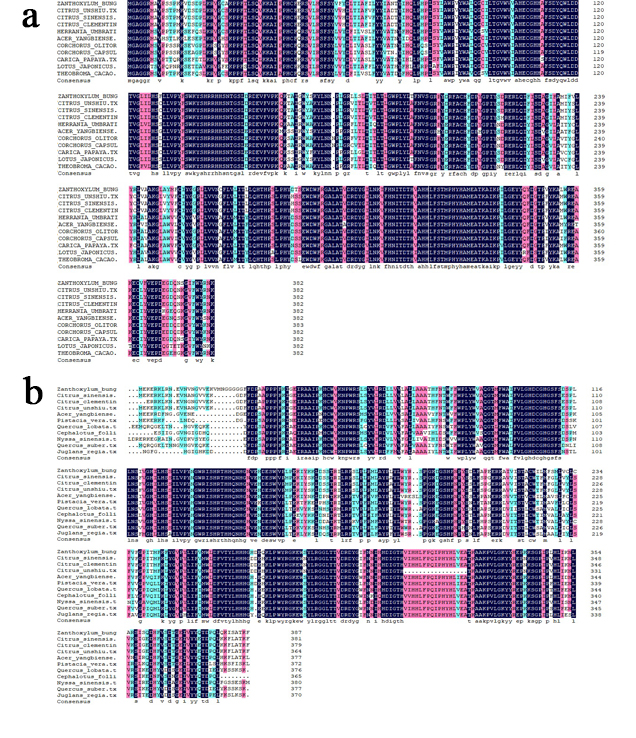


**Figure S3.** Alignment of the *FAD2* and *FAD3* amino acid sequences of different plant species.


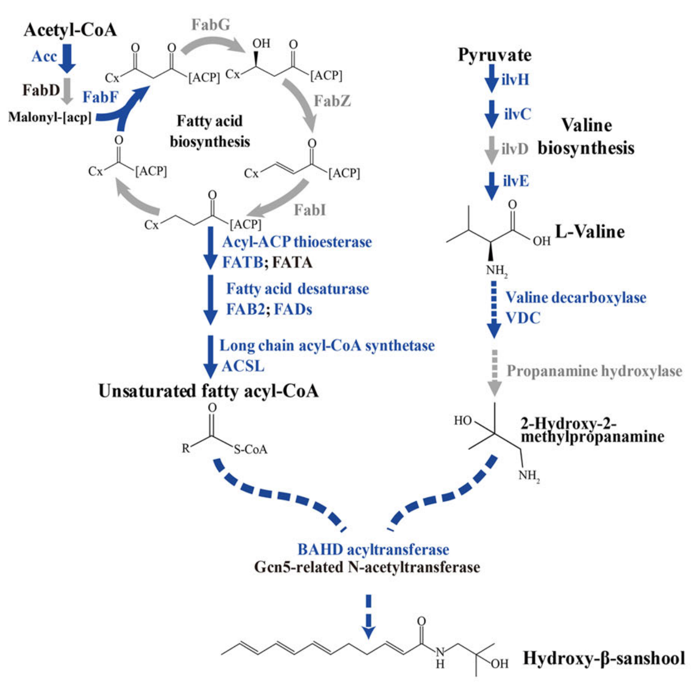


**Figure S4.** A Schematic representation of sanshool biosynthesis pathways. The solid lines indicate genes catalyzing major reactions that were characterized. The dotted lines indicate unclear pathways. The blue color indicates the species-specific expanded genes in Z. bungeanum.

**Table S1.** Sequences of the primers used for the candidate genes.

| Primer | Sequence | Tm (°C) |
| --- | --- | --- |
| **Amplification** |  |  |
| ZbFAD2-F  ZbFAD2-R | ggggtaccATGGGTGCAGGTGGACG  cgggatccTCAAATCTTATTTCTGTACCAGAAGA | 55 |
| ZbFAD3-F ZbFAD3-R | ggggtaccATGGAGAAAGAAAGAAAGTTGAGAAA  cgggatccTCATTCAAATTTGGTTGCCGAAATTT | 56 |
| **qRT-PCR** |  |  |
| ZbFAD2-F  ZbFAD2-R | TCCTCCACTCATTCCT  GCCTGCCTGATACATT | 56 |
| ZbFAD3-F ZbFAD3-R | TTTTCGTAATGTGGTTGG  ATAATGCGGGATTTGAGG | 56 |
| β-actin-F  β-actin-R | GTGCTGGATTCTGGTGATGG  ATTTCCCGTTCGGCTGTG | 56 |
